# Survey of Zoonotic Diarrheagenic Protist and Hepatitis E Virus in Wild Boar (*Sus scrofa*) of Portugal

**DOI:** 10.3390/ani13020256

**Published:** 2023-01-12

**Authors:** Sérgio Santos-Silva, Danny Franciele da Silva Dias Moraes, Pedro López-López, Josman D. Palmeira, Rita T. Torres, Maria São José Nascimento, Alejandro Dashti, David Carmena, Antonio Rivero-Juarez, João R. Mesquita

**Affiliations:** 1 School of Medicine and Biomedical Sciences (ICBAS), University of Porto, 4050-313 Porto, Portugal; 2 State Department for the Environment of Mato Grosso (SEMA), Cuiabá 78049-913, Brazil; 3 Unit of Infectious Diseases, Hospital Universitario Reina Sofia, Clinical Virology and Zoonoses, Instituto Maimonides de Investigación Biomédica de Córdoba (IMIBIC), Universidad de Córdoba (UCO), 14004 Cordoba, Spain; 4 CESAM & Department of Biology, University of Aveiro, Campus de Santiago, 3810-193 Aveiro, Portugal; 5 Faculty of Pharmacy, University of Porto, 4050-313 Porto, Portugal; 6 Parasitology Reference and Research Laboratory, National Centre for Microbiology, 28220 Madrid, Spain; 7 Center for Biomedical Research Network (CIBER) in Infectious Diseases, Health Institute Carlos III, 28220 Madrid, Spain; 8 Epidemiology Research Unit (EPIUnit), Instituto de Saúde Pública da Universidade do Porto, 4050-600 Porto, Portugal; 9 Laboratory for Integrative and Translational Research in Population Health (ITR), 4050-600 Porto, Portugal

**Keywords:** *Cryptosporidium* spp., *Balantioides coli*, *Blastocystis* sp., hepatitis E virus, zoonotic, emerging infectious diseases

## Abstract

**Simple Summary:**

Enteropathogenic viruses, such as hepatitis E virus, and diarrhoeagenic protists have been frequently reported in swine and can infect a wide range of mammals, including humans. Data on their fecal shedding and circulation pathways are still lacking or incomplete. Hence, the aim of the present study was to characterize the presence of microeukaryotes and HEV in the wild boar of Portugal. Of the 144 samples tested, 2 showed the presence of *Cryptosporidium scrofarum*, 21 *Balantioides coli*, 42 *Blastocystis* ST5, and 4 HEV genotype 3. The present work shows that potentially zoonotic protozoa and HEV are circulating in wild boar populations in Portugal.

**Abstract:**

Enteropathogenic parasites and viruses have been frequently reported in swine and can infect a wide range of mammals, including humans. Among the wide variety of parasites infecting swine, diarrhoeagenic protists are among those that cause significant morbidity. Hepatitis E virus (HEV) has also been reported both in domestic pigs and wild boar and is known to have an important public health significance. These agents share the fecal–oral transmission route, but data on their fecal shedding and circulation pathways are still lacking or incomplete. Hence, the aim of the present study was to characterize the presence of microeukaryotes and HEV in the wild boar of Portugal. Wild boar stool samples (*n* = 144) were obtained during the official hunting seasons (October to February) in 2018/2019, 2019/2020, and 2021/2022 and tested for *Cryptosporidium* spp., *Balantioides coli*, *Giardia duodenalis*, *Blastocystis* sp., *Enterocytozoon bieneusi* and HEV by molecular assays, followed by sequencing and phylogenetic analysis. We have detected *Cryptosporidium scrofarum* (1.4%, 95% CI: 0.2–4.9), *B. coli* (14.6%, 95% CI: 9.2–21.4), *Blastocystis* ST5 (29.2%, 95% CI: 21.9–37.2) and HEV genotype 3 (2.8%, 95% CI: 0.7–6.9; subgenotypes 3e and 3m). Co-infections were observed in thirteen animals where two were positive for both HEV and *B. coli*, one was positive for both *C. scrofarum* and *Blastocystis* ST5, and ten were positive for both *B. coli* and *Blastocystis* ST5. *Giardia duodenalis* and *E. bieneusi* were not detected in the surveyed wild boar population. As far as we know, this is the first report describing protist infections by *Cryptosporidium* spp., *B. coli*, and *Blastocystis* sp., as well as the first identification of the emerging HEV genotype 3m in wild boar of Portugal. The present work shows that potentially zoonotic protozoa and HEV are circulating in wild boar populations in Portugal. Awareness and epidemic-surveillance network implementation measures targeting wild boar are needed to prevent the spread of these pathogenic agents to humans.

## 1. Introduction

The presence of zoonotic parasites and viruses has been frequently reported in swine and is a potential threat to human health [1,2]. Animals can host a variety of parasites, such as those in the *Cryptosporidium*, *Balantioides*, *Giardia*, *Blastocystis*, and *Enterocytozoon* genera, that may have a negative impact on their welfare [3,4,5,6,7]. Among the wide variety of parasites infecting swine, diarrhoeagenic protists are known to cause significant morbidity in wild boar and domestic pigs [8,9,10]. Direct contact with infected hosts or their fecal material, or indirect transmission through the consumption of contaminated water or food, are the most common transmission pathways to animals and humans [5,11,12,13,14,15,16,17,18,19,20,21]. Of those, *Cryptosporidium* spp. infections have a direct impact on development rates and are linked to significant economic losses in livestock production [22,23]. Up until now, at least 46 *Cryptosporidium* species have been identified [24,25], of which host-adapted *C. suis* and *C. scrofarum*, and zoonotic *C. parvum*, *C. felis*, *C. muris*, *C. tyzzeri*, and *C. andersoni* have been reported in pigs [26,27,28,29,30]. *Cryptosporidium suis* seems to be more prevalent among pre-weaned pigs, whereas *C. scrofarum* is among starters, especially those weaned at a younger age [27]. *Balantioides coli*, formerly known as *Balantidium coli*, is a protozoan ciliate able to infect pigs, cattle, sheep, goats, camels, equids, and human and non-human primates [9]. Domestic pigs and wild boars are the dominant reservoir host species. Of note, *B. coli* is regarded as the only ciliate known to infect humans [31]. Most swine and human infections by this protozoan are asymptomatic [9]. To date, three *B. coli* genotypes (A, B, and C) have been identified. Genotypes A and B have mostly been found in pigs, whereas genotype C is prevalent in non-human primates [32].

*Giardia duodenalis* is a parasite that affects humans and many other animals. This protozoan has two morphological stages, trophozoite and cyst, where the latter is the infective stage. *Giardia duodenalis* is now classified as a species complex with eight (A–H) distinct genotypic assemblages. In domestic pigs, assemblage E is usually the most common assemblage found [33,34], although assemblages A, B, C, D, and F have also been sporadically detected [35,36].

The non-flagellated stramenopile *Blastocystis* sp. is commonly found in the intestine of humans and a wide spectrum of animals [37]. It is regarded as the most common eukaryotic agent present in human feces [38]. *Blastocystis* sp. has a high degree of genetic variability at the small subunit of the ribosomal RNA gene (*ssu* rDNA) [39,40], with 30 proposed subtypes (STs) [41,42,43]. ST1-ST8 and ST12 have the significant zoonotic potential [44]. Initially thought to be human-specific, ST9 has also been identified in peafowl [45] and non-human primates [46], among other animals’ species. Additionally, ST10, ST14, and ST23 have also been described in humans [47,48]. The remaining subtypes have only been isolated from non-human animal species [43,45,49,50,51,52,53,54].

*Enterocytozoon bieneusi* is a fungi-related, obligate intracellular pathogen that infects intestinal epithelial cells, resulting in severe or chronic diarrhea and malabsorption [55]. Over 600 genotypes of *E. bieneusi* have been reported and classified into 11 phylogenetic groups based on polymorphisms in the internal transcribed spacer (ITS) of the *ssu* rRNA gene [56,57]. Host-adapted genotypes are found in groups 3–11, whereas zoonotic genotypes reported in humans and animals fall within groups 1 and 2 [58]. Pigs are the primary reservoir host for *E. bieneusi* [3]. Currently, 134 ITS *E. bieneusi* genotypes have been identified in pigs or wild boars worldwide [59]. Among them, 19 genotypes (BEB4, CAF1, CS-1, CS-4, D, EbpA, EbpC, EbpD, H, Henan-III, Henan-IV, I, LW1, O, PigEBITS5, PigEBITS7, PigEB10, and SH8) have also been detected in humans and are therefore considered zoonotic [59]. More than 20 genotypes have been identified in wild boars, the majority of which (D, EbpA, EbpC) are zoonotic [58].

Besides parasites, viruses also frequently infect animals and humans [2]. Among them, the hepatitis E virus (HEV) is regarded as an emerging public health concern [60]. Hepatitis E virus belongs to the *Hepeviridae* family, genus *Paslahepevirus* [61,62,63], and causes hepatitis in humans by consumption of undercooked meat and meat products in industrialized countries [64].

HEV genotypes 3–7 are zoonotic [65], with viral strains being isolated from both human and animal populations. The main HEV reservoirs are domestic pigs and wild boar [66,67], but zoonotic strains have also been found in rabbits, deer, camels, and rats [68]. Most HEV infections in developed countries are autochthonous, caused by HEV 3, and spread via zoonotic HEV infection from raw or undercooked swine meat or direct contact with infected swine [68]. In Europe, HEV 3 is the predominant genotype, and those strains belonging to the clade 3efg are thought to be associated with more severe illnesses [69].

With over 230,000 licensed hunters currently, the Portuguese population has a long tradition of hunting and eating game meat (Relatório de Actividade Cinegética, 2022). In Portugal, HEV 3 has been detected in hunted wild boar and domestic pigs [70,71]. The HEV 3 circulation in swine likely contributed to the 16.3% anti-HEV IgG seroprevalence detected in a nationwide Portuguese population serosurvey [72]; however, molecular information on circulating HEV in Portugal is still scarce.

Studies on microeukaryotes and viruses have been increasing over time [1,73,74,75]. However, there is still an important lack of information on co-infections involving both microeukaryotes and HEV in wild boar, with recent data suggesting an interaction between these two groups of enteric pathogens [1]. The aim of the present study was to detect and characterize the circulation of microeukaryotes and HEV in the wild boar of Portugal.

## 2. Materials and Methods

### 2.1. Sample Collection

**Figure 1:**
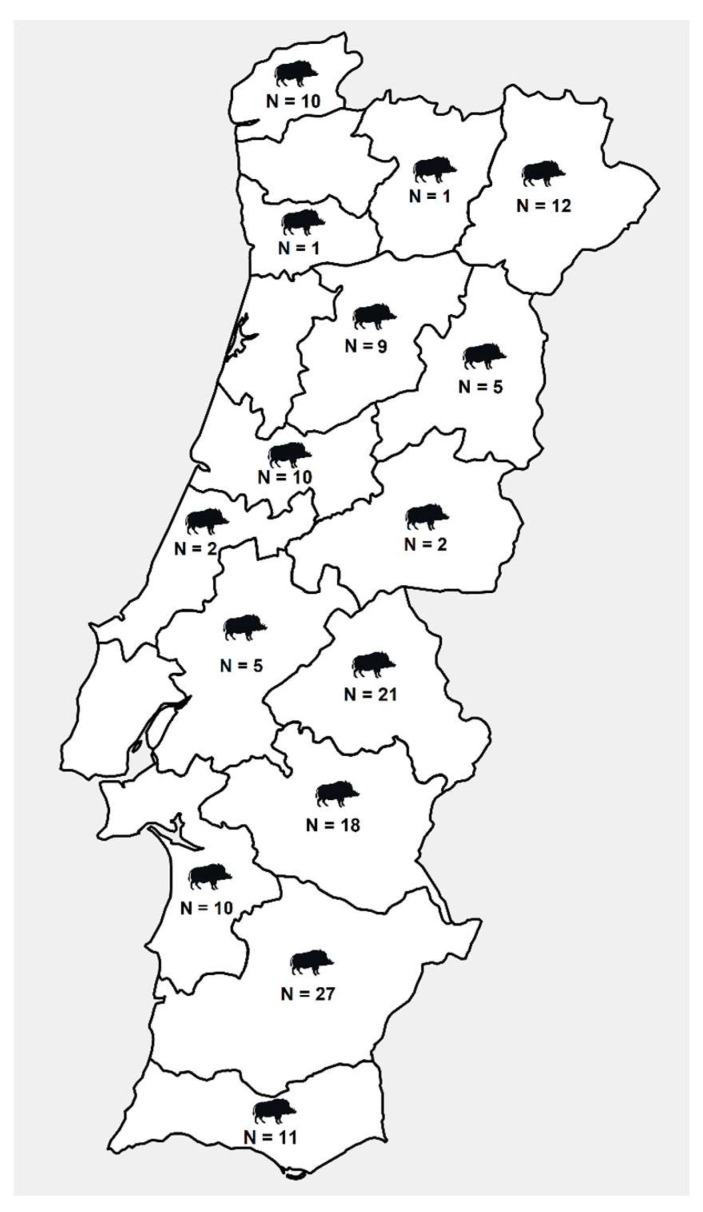
Geographical distribution of wild boar samples in Portugal.

Individual wild boar stool samples (*n* = 144) were obtained during three official hunting seasons (October to February) in 2018/2019, 2019/2020, and 2021/2022 across Portugal (Figure 1). Within 1–3 h after death, fecal samples were collected from the posterior part of the large intestine. All fecal samples processed and tested in the present study were formed, suggestive of light pathogen infections and absence of gastrointestinal manifestations (diarrhea). No animals were killed for the sake of this study. All stool samples were kept at 4 °C and transported to the lab within 12 h. Samples were then stored at −20 °C until DNA/RNA extraction, which was completed within 2 weeks of collection.

### 2.2. DNA and RNA Extraction

Fecal suspensions (10%) were prepared in phosphate-buffered saline pH 7.2 and centrifuged for 5 min at 8000× *g*. DNA and RNA were simultaneously extracted and purified using the QIAamp Cador Pathogen Mini Kit (Qiagen, Hilden, Germany), according to the manufacturer’s instructions using 200 µL of the clarified supernatants in the QIAcube® automated platform (Qiagen). Eluted DNA and RNA were stored at −80 °C with RNase-free water. 

### 2.3. Molecular Detection of Cryptosporidium *spp.*

To detect *Cryptosporidium* spp., a nested-PCR assay was used to amplify a 587 bp fragment of the *ssu* rRNA gene with the primer sets CR-P1/CRP2 and CR-P3/CPB-DIAGR, as recommended by Tiangtip and Jongwutiwes (2002) [76].

### 2.4. Molecular Detection of Balantioides coli

Detection of *B. coli* was attempted by a direct PCR assay targeting the complete ITS1–5.8s-rRNA–ITS2 region and the last 117 bp (3t’ end) of the *ssu* rRNA gene (400 bp) using the primer set B5D/RD5 as proposed by Ponce-Gordo et al. (2011) [77].

### 2.5. Molecular Detection of Giardia duodenalis

For the detection of *G. duodenalis* a real-time PCR (qPCR) assay was used to amplify a 62-bp region of the *ssu* rRNA gene with the primer set Gd-80F/Gd-127R as proposed by Verweij et al. (2003) [78].

### 2.6. Molecular Detection of Blastocystis *sp.*

For the detection of *Blastocystis* sp., a direct PCR assay was used to amplify a 600-bp region of the *ssu* rRNA gene with the pan-*Blastocystis*, barcode primer set RD5/BhRDr according to Scicluna et al. (2006) [79].

### 2.7. Molecular Detection and Characterization of Enterocytozoon bieneusi

For the detection of *E. bieneusi*, a nested PCR assay was used to amplify the ITS region as well as sections of the surrounding large and small subunits of the rRNA gene (390 bp) with the primer sets EBITS3/EBITS4 and EBITS1/EBITS2.4 as described by Buckholt et al. (2002) [80].

### 2.8. Molecular Detection of HEV

For the detection of HEV, a broad nested RT-PCR assay was used targeting the RNA-dependent RNA-polymerase (*RdRp*) gene of the open reading frame ORF 1 region of the genome (331–334 bp) with the primer sets HEV-cs/HEV-cas and HEV-csn/HEV-casn (Johne et al., 2010) [81].

### 2.9. General Procedures

Oligonucleotides used for the molecular detection of the parasites and HEV described above are shown in Table S1. All qPCR reactions were run on a CFX Connect Real-Time PCR Detection System (Bio-Rad; Hercules, CA, USA). All direct and nested PCR reactions were run on a T100 thermocycler (Bio-Rad). Reaction mixtures included Fast qPCR Mastermix (Probe) (GriSP^®^, Porto, Portugal), Fast PCR Mastermix (GriSP^®^), and 2x Xpert Fast Hotsart Mastemix (GriSP^®^). The amplified DNA fragments were identified by electrophoresis of PCR amplification products at 100 V for 40 min on 1.5% agarose gels stained with Xpert Green Safe DNA gel dye (GriSP®), and UV light was irradiated to confirm the results.

### 2.10. Sequencing and Phylogenetic Analysis

Using GRS PCR & Gel Band Purification Kit (GriSP®), amplicons that appeared to be positive and the expected size were purified. After purification using the Sanger method and the correct internal specific primers for the target gene, bidirectional sequencing was carried out, and with the help of the BioEdit Sequence Alignment Editor v7.1.9 software package, version 2.1, sequences were aligned and compared to sequences found in the NCBI (GenBank) nucleotide database, which were retrieved on 3 November 2022 (http://blast.ncbi.nlm.nih.gov/Blast). MEGA version X software [82] and the Interactive Tree Of Life (iTOL) platform [83] were used for the phylogenetic analysis, together with the sequences found in this work and additional representative sequences received from GenBank. The maximum-likelihood (ML) approach was used to infer this analysis [82,84], and Tamura 3-parameter model was used to estimate the ML bootstrap values using 1000 replicates [84]. This model was determined by MEGA version X [82] to be the most effective replacement. The sequences obtained in this study were deposited in GenBank with accession numbers OM349065-OM349073/OP779215-OP779226 (Balantioides coli), ON322862-ON322864/ON322866-ON322871/OP765595-OP765627 (Blastocystis), OM319689-OM319690 (Cryptosporidium scrofarum) and OM751885-OM751887/OP765582 (HEV).

### 2.11. Statistical Analysis

The prevalences of microeukaryote parasites and HEV in wild boar of Portugal populations investigated were calculated based on the ratio of the number of positive samples to the total number of samples examined with a 95% confidence interval (95% CI). Statistical analysis of association in co-infections among microeukaryote parasites and HEV was conducted using the Chi-square (χ^2^) or Fisher’s exact test performed with GraphPad Prism 5.0 (GraphPad Software, Inc., San Diego, CA, USA). A *p*-value ≤ 0.05 was used to determine that the observed differences were statistically significant.

## 3. Results

**Figure 2:**
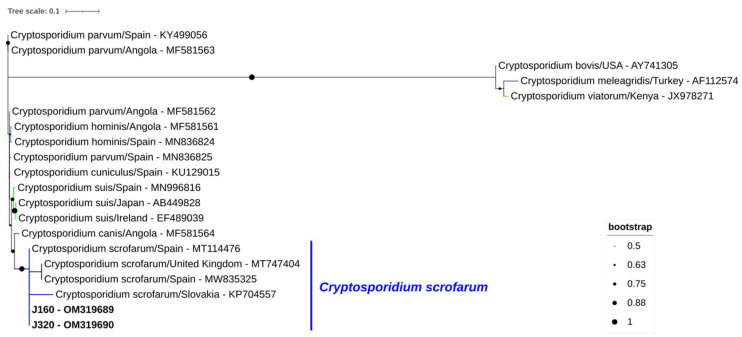
Phylogenetic analysis of *Cryptosporidium* found in wild boars. Tree inferred using the MEGA X maximum likelihood method (Tamura 3-parameter model) and the Interactive Tree of Life (iTOL) based on 20 nucleotide *Cryptosporidium* spp. sequences at the *ssu* rRNA marker, including sequences found in this study (*Cryptosporidium scrofarum*, plus its accession number, is in bold) and 18 strains of different *Cryptosporidium* species obtained from GenBank (no bold or shading and identified with the accession number and its species and country of origin).

*Cryptosporidium* spp. was detected in two out of 144 stool samples of wild boar (1.4%; 95% confidence interval [CI]: 0.2–4.9) (Table 1). Sequence analyses of the obtained amplicons showed 100% identity with a *C. scrofarum* sequence from a wild boar in neighboring Spain (MT114476) (Figure 2).

**Figure 3:**
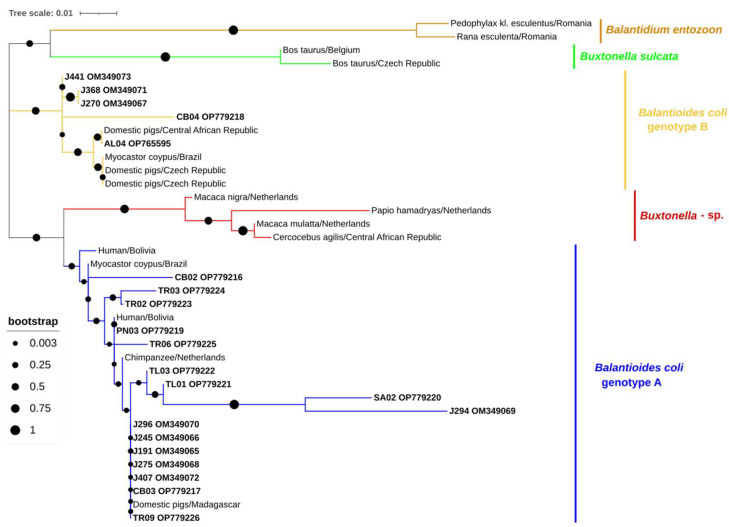
Phylogenetic analysis of *Balantioides coli* found in wild boars. Tree inferred using the MEGA X maximum likelihood method (Tamura 3-parameter model) and the Interactive Tree of Life (iTOL) based on 38 nucleotide *Balantioides coli* sequences at the ITS marker, including sequences found in this study (*Balantioides coli* genotypes A and B, plus its accession numbers, is in bold) and 17 strains of different genotypes obtained from GenBank (no bold or shading and identified with the accession number, host and country of origin).

Twenty-one samples tested positive for *B. coli* (14.6%, 95% CI: 9.2–21.4). Six samples generated sequences showing 95–100% identity with a *B. coli* isolate from a pig in South Korea (MZ676851); two shared 99.6% and 98.8% identity, respectively, with a *B. coli* isolate also from a South Korean pig (MZ676842), one sequence shared 100% identity with a *B. coli* isolate from a pig in Malaysia (MG734707), one shared 96.8% identity with a *B. coli* isolate from a pig in Kenya (JQ073378), three sequences shared 99.4–100% identity with a *B. coli* isolate from a pig in China (MT252055), one shared 100% identity with a *B. coli* isolate from a pig in Spain (MW648345), four sequences shared 99.4–100% identity with a *B. coli* isolate from a pig in China (MT252069), one shared 98.5% identity with a *B. coli* isolate from a pig in China (MT252062), one shared 97.4% identity with a *B. coli* isolate from a pig in Spain (MT112069), and one shared 100% identity with a *B. coli* isolate from a pig in China (MT252079). Sixteen and five *B. coli* sequences were assigned to genotypes A and B, respectively (Figure 3).

**Figure 4:**
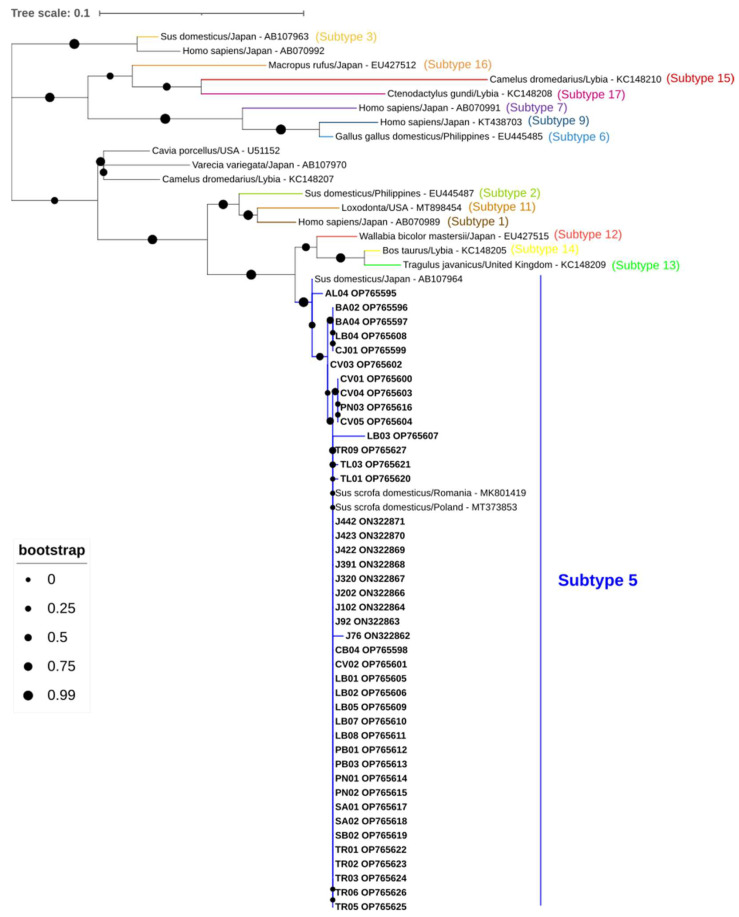
Phylogenetic analysis of *Blastocystis* found in wild boars. Tree inferred using the MEGA X maximum likelihood method (Tamura 3-parameter model) and the Interactive Tree of Life (iTOL) based on 62 nucleotide *Blastocystis* sequences at the *ssu* rRNA marker, including sequences found in this study (*Blastocystis* subtype 5, plus its accession numbers, is in bold) and 20 strains of different subtypes obtained from GenBank (subtype 1-17) (no bold or shading and identified with the accession number, host and country of origin).

*Blastocystis* sp. was identified in 42 samples (29.2%, 95% CI: 21.9–37.2). BLAST analysis indicated that six sequences shared 99.8–100% identity with a *Blastocystis* sp. isolated from a pig in Romania (MK801419), one shared 100% identity with a *Blastocystis* sp. isolated from a domestic pig in Romania (MK801418), and thirty-five shared 98.8–100% identity with *Blastocystis* isolate from a pig in Poland (MT373853). All *Blastocystis* sequences were identified as subtype 5 (Figure 4).

**Figure 5:**
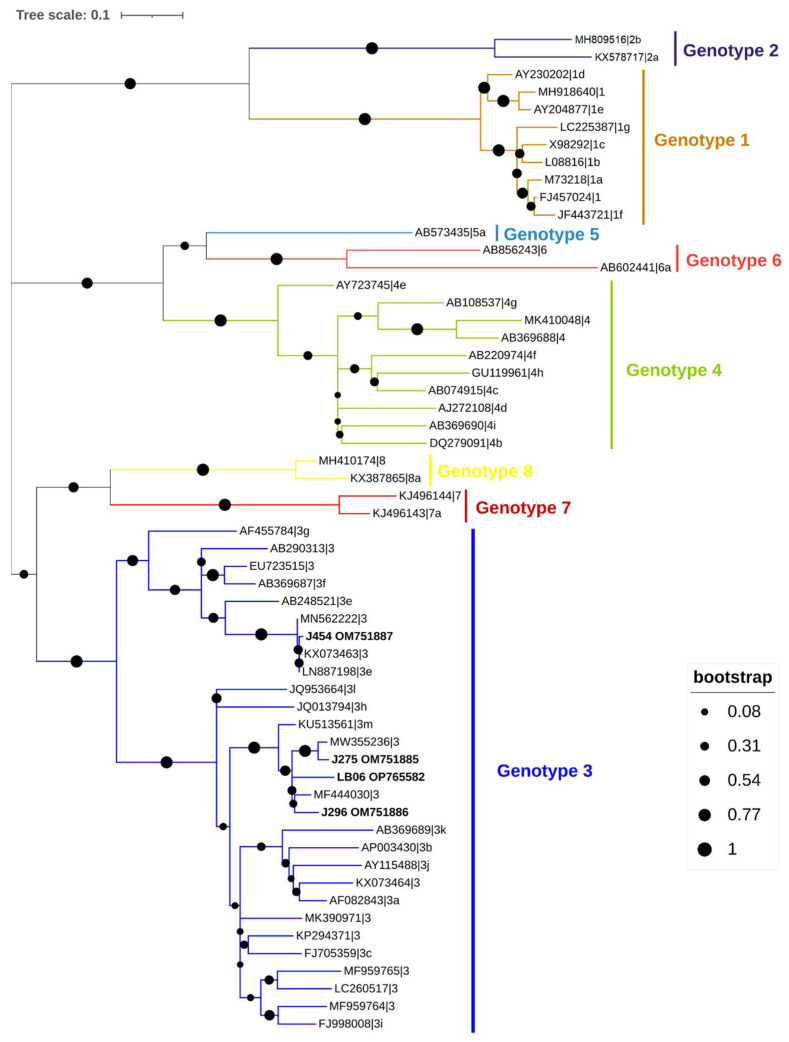
Phylogenetic analysis of HEV found in wild boars. Tree inferred using the MEGA X maximum likelihood method (General Time Reversible model) and the Interactive Tree of Life (iTOL) based on 57 nucleotide HEV sequences at the RNA-dependent RNA-polymerase, including sequences found in this study (HEV-3, plus its accession number, is in bold) and 53 strains of different genotypes obtained from GenBank (HEV-1 to HEV-8) (no bold or shading and identified with the accession number and its genotype and subgenotype).

Four samples were positive for HEV (2.8%, 95% CI: 0.7–6.9). Amplicons from two samples generated sequences sharing 94.1% and 97.0% identity with human isolates from France (MW355236 and MF444030, respectively), one sequence shared 99.3% identity with an isolate from a mussel in Spain (LN887198), and one shared 96.2% identity with a human isolate from Spain (MZ289103). All HEV sequences belonged to genotype 3, being three of subtype 3m and one subtype 3e (Figure 5).

Molecular testing for the presence of *G. duodenalis* and *E. bieneusi* yielded negative results.

Co-infections were observed in thirteen animals (Table 2 and Table S2). Two were positive for both HEV and *B. coli*, one was positive for both *C. scrofarum* and *Blastocystis* ST5, and ten were positive for both *B. coli* and *Blastocystis* ST5. In this study, no association between *Cryptosporidium* spp., *Balantioides coli*, *Giardia duodenalis*, *Blastocystis* sp., *Enterocytozoon bieneusi,* and HEV was significant (Table S3).

## 4. Discussion

Here, we report the first description of protist infections by *Cryptosporidium* spp., *B. coli*, and *Blastocystis* sp., as well as the first identification of the emerging HEV genotype 3 m in the wild boar of Portugal.

*Cryptosporidium* infections were detected at a 1.4% prevalence. The occurrence rates of this pathogen in other European wild boar populations varied largely from 8.2% (Spain, [1]), 13.3% (Central Europe, [27]), and 25% (Sweden, [29]). However, care should be taken when comparing prevalence data from different studies, as different detection methods (microscopy, PCR) differ in diagnostic sensitivities. Molecular methods are considered a highly sensitive and specific analytical tool for the detection and characterization of infections, providing reliable data when compared to conventional parasitological methods [85]. Notwithstanding, the performance of PCR-based methods can be affected by several factors, including inefficient removal of inhibitors present in stools, low parasitic load, and insufficient quantity/quality of starting DNA material, potentially leading to underestimated prevalence rates [86]. The *B. coli* prevalence of 14.6% found in the wild boar population studied was slightly higher than that from a study conducted in neighboring Spain, where an 11.7% prevalence rate was reported [1]. Interestingly, a recent study has identified *B. coli* in the wild cervids of Portugal [87], suggesting potentially different novel transmission routes. Nevertheless, the discrepancies observed in prevalence between the present study and the study mentioned before conducted in Spain may be explained, at least partially, by epidemiologic factors, as samples from the study were retrospectively studied up to 20 years ago.

*Blastocystis* sp. was also detected in this wild boar population, with an occurrence rate of 29.2%. As far as we know, this is the first study reporting the presence of *Blastocystis* sp. in wild boar from Portugal. Additionally, our results showed a lower prevalence of the agent than compared with one from Italy (61.9%, [88]) and substantially higher than a similar study conducted in Spain (0.1%, [1]). Nevertheless, these prevalence rate differences should be analyzed with care as the sample size from Italy was considerably lower than the present study, and the sample size from the study conducted in Spain was similar, which shows a higher circulation of *Blastocystis* in wild boar of Portugal when comparing to those of the study from Spain. Furthermore, our molecular data confirm host-adapted ST5 as the main *Blastocystis* genetic variant circulating in European wild boar.

Remarkably, neither the protozoan *G. duodenalis* nor the microsporidia *E. bieneusi* were detected. Both pathogens have been previously described, with occurrences ranging from 1.7–2.1% in Croatia [89] and Spain [3]. Our lack of positive results for these two species may be related to the relatively low sample size analyzed in the present study.

Concerning viral detection, the overall occurrence of HEV (2.8%) was lower than that previously reported in a Portuguese study (10%) on wild boar stools [71]. The HEV sequences detected in wild boar were of subgenotype 3e (J454) and subgenotype 3m (J275; J296; LB06) [90]. Subgenotype 3e has been found in wild boars in Portugal and Germany, while countries such as France, Italy, Slovenia, Sweden, and the United Kingdom reported HEV 3e in pigs [91]. In Europe, the predominant HEV genotype is HEV 3, with severe illness being associated with those strains belonging to clade 3efg [69]. Of note, HEV 3 found in our study has been linked to fulminant autochthonous hepatitis E in humans in Europe [92]. Interestingly, subgenotype 3m has been detected in three wild boars from this study. This subgenotype was recently discovered in the same host species in southern Spain [93], having also been detected in humans of the same region. Our study is the first to report the detection of this genotype in Portugal that belongs to the same clade as other sequences recently isolated from humans in France and Spain. Since zoonotic transmission of this novel HEV 3 subgenotype has already been demonstrated [94], more surveys should be conducted in human populations.

In this study, protist and HEV co-infections were detected in thirteen wild boars (Table 2). One sample had a concomitant infection by *Blastocystis* ST5 and *C. scrofarum*, two samples were co-infected with *B. coli* genotype A and HEV genotype 3m, and ten samples were co-infected with *B. coli* and *Blastocystis* ST5. Previous reports have suggested that enteroparasites in co-infection with HEV can modulate the infectivity of the latter in swine [1,75]. That survey hypothesized that the presence of extracellular *G. duodenalis* and *Blastocystis* sp. could offer protection against HEV infection by an unknown mechanism, whereas intracellular *Cryptosporidium* spp. and *E. bieneusi* had the reverse effect, favoring HEV infection [1,75]. Additionally, there are two mechanisms that can explain the interaction between viruses and enteroparasites, such as mechanical competition or cross-immune impairment. The effect of HEV infection increased sensitivity in the case of intracellular enteroparasites may be connected to the enteroparasites’ induction of immune evasion. However, extracellular enteroparasites may potentially have an immune-related mechanism that reduces the vulnerability to HEV infection [75]. Our results show that there is no association between the presence of the microeukaryote parasites reported in this study and HEV. Nevertheless, caution should be taken when interpreting our results as the number of positive animals is low. The above-mentioned associations would require additional studies involving larger sample sizes.

Additionally, the prevalence rates of the microeukaryotes *Blastocystis* and *B. coli* were higher in the hunting season of 2021/2022 (42.9% and 15.6%, respectively) when compared with the previous 2018/2019 (30% and 0%) and 2019/2020 (10.3% and 15.5%) seasons, with positive samples being widely distributed throughout the country in all studied seasons, showing an increase in cases during these past few years. Moreover, *C. scrofarum* was only detected in 2 wild boar samples from the hunting season of 2019/2020 in the central area of Portugal. Finally, HEV was not detected in samples from the hunting season of 2018/2019, whilst three samples from the hunting season 2019/2020 were positive for HEV with subgenotypes 3e and 3m being described, and one from 2021/2022 with subgenotype 3m characterized which was collected in the same district (Viana do Castelo) as the subgenotype 3e identified in 2019/2020, showing the potential circulation of several subgenotypes of HEV genotype 3 in wild boar of this region.

## 5. Conclusions

In conclusion, the present work shows that potentially zoonotic protozoa and virus are circulating in wild boar populations in Portugal. Awareness and implementation of an epidemic-surveillance network targeting wild ungulates should be carried out to prevent the spread of these pathogenic agents to humans. To avoid the dissemination of zoonotic agents, veterinarians, and hunters, also meat suppliers and consumers must establish a comprehensive approach to biosecurity and awareness.

## Figures and Tables

**Table 1:** Summary of prevalences of microeukaryote parasites and HEV in wild boar of Portugal.

	*Cryptosporidium* spp.	*Balantioides coli*	*Giardia duodenalis*	*Blastocystis* spp.	*Enterocytozoon bieneusi*	Hepatitis E Virus
Positive samples	2	21	0	42	0	4
Prevalence	2/144 (1.4%)	21/144 (14.6%)	0/144 (0%)	42/144 (29.2%)	0/144 (0%)	4/144 (2.8%)

**Table 2:** Summary of pathogen detection in stools.

Fecal Presence	No. of Samples
*Balantioides coli* + *Blastocystis* spp.	10
*Balantioides coli* + Hepatitis E virus	2
*Blastocystis* spp. + *Cryptosporidium* spp.	1
*Blastocystis* spp.	42
*Balantioides coli*	21
Hepatitis E virus	4
*Cryptosporidium* spp.	2
*Giardia duodenalis*	0
*Enterocytozoon bieneusi*	0

## Data Availability

The data presented in this study are available on request from the corresponding author.

## Supplementary Materials

The following supporting information can be downloaded at: https://www.mdpi.com/article/10.3390/ani13020256/s1, Table S1: Oligonucleotides used for the molecular identification and/or characterization of the microeukaryote parasites and HEV investigated in the present study; Table S2: Summary of positive samples for microeukaryote parasites and HEV from wild boar of Portugal; Table S3: Summary of co-infections between microeukaryote parasites and HEV and respective *p*-value from wild boar of Portugal.
