# Supplementary material for: Survey of Zoonotic Diarrheagenic Protist and Hepatitis E Virus in Wild Boar (Sus scrofa) of Portugal
Source: Animals (Basel). 2023 Jan 12;13(2):256. doi: 10.3390/ani13020256 (PMC9854796; doi:10.3390/ani13020256)
Supplement: Supplementary file 1 [file animals-13-00256-s001.zip › animals-2132670-supplementary.pdf]

Table S1. Oligonucleotides used for the molecular identification and/or characterization of the microeukaryote parasites and HEV investigated in the present study.

Target organism	Locus	Oligonucleotide	Sequence (5′–3′)	Reference
Cryptosporidium spp.	*ssu* rRNA	CR-P1	CAGGGAGGTAGTGACAAGAA	[76]
		CR-P2	TCAGCCTTGCGACCATACTC	[76]
		CR-P3	ATTGGAGGGCAAGTCTGGTG	[76]
		CPB-DIAGR	TAAGGTGCTGAAGGAGTAAGG	[76]
Balantioides coli	ITS	B5D	GAGCTTTTTAACTGCAACAACG	[77]
		RD5	ATCTGGTTGATCCTGCCAGT	[77]
Giardia duodenalis	*ssu* rRNA	Probe	FAM–CCCGCGGCGGTCCCTGCTAG–BHQ1	[78]
		Gd-80F	GACGGCTCAGGACAACGGTT	[78]
		Gd-127R	TTGCCAGCGGTGTCCG	[78]
Blastocystis sp.	*ssu* rRNA	BhRDr	GAGCTTTTTAACTGCAACAACG	[79]
		RD5	ATCTGGTTGATCCTGCCAGT	[79]
Enterocytozoon bieneusi	ITS	EBITS3	GGTCATAGGGATGAAGAG	[80]
		EBITS4	TTCGAGTTCTTTCGCGCTC	[80]
		EBITS1	GCTCTGAATATCTATGGCT	[80]
		EBITS2.4	ATCGCCGACGGATCCAAGTG	[80]
HEV	ORF1	HEV-cs	TCGCGCATCACMTTYTTCCARAA	[81]
		HEV-cas	GCCATGTTCCAGACDGTRTTCCA	[81]
		HEV-csn	TGTGCTCTGTTTGGCCCNTGGTTYCDG	[81]
		HEV-casn	CCAGGCTCACCRGARTGYTTCTTCCA	[81]

ITS: Internal transcribed spacer; *ssu* rRNA: Small subunit ribosomal RNA; ORF: Open reading frame.

Table S2. Summary of positive samples for microeukaryote parasites and HEV from wild boar of Portugal.

Sample ID	Sex	Age	*Cryptosporidium scrofarum*	*Balantioides coli*	*Giardia duodenalis*	*Blastocystis*	*Enterocytozoon bieneusi*	HEV
J76	Female	Adult	-	-	-	+	-	-
J92	Female	Young Adult	-	-	-	+	-	-
J102	Male	Young Adult	-	-	-	+	-	-
J160	Male	Young	+	-	-	-	-	-
J191	Male	Young	-	+	-	-	-	-
J202	Female	Adult	-	-	-	+	-	-
J245	Female	Adult	-	+	-	-	-	-

J270	Female	Adult	-	+	-	-	-	-
J275	Male	Adult	-	+	-	-	-	+
J294	Male	Young	-	+	-	-	-	-
J296	Female	Adult	-	+	-	-	-	+
J320	Male	Young	+	-	-	+	-	-
J368	Female	Adult	-	+	-	-	-	-
J391	Female	Young	-	-	-	+	-	-
J407	Female	Adult	-	+	-	-	-	-
J422	Female	Adult	-	-	-	+	-	-
J423	Male	Adult	-	-	-	+	-	-
J441	Female	Adult	-	+	-	-	-	-
J442	Male	Adult	-	-	-	+	-	-
J454	Female	Young	-	-	-	-	-	+
CV-01	Male	Young	-	-	-	+	-	-
CV-02	Male	Adult	-	-	-	+	-	-
CV-03	Male	Young	-	-	-	+	-	-
CV-04	Male	Young	-	-	-	+	-	-
CV-05	Male	Young	-	-	-	+	-	-
SA-01	Female	Adult	-	-	-	+	-	-
SA-02	Female	Young	-	+	-	+	-	-
PB-01	Male	Young	-	-	-	+	-	-
PB-03	Female	Adult	-	-	-	+	-	-
PN-01	Male	Young	-	-	-	+	-	-
PN-02	Female	Adult	-	-	-	+	-	-
PN-03	Female	Adult	-	+	-	+	-	-
CJ-01	Male	Adult	-	-	-	+	-	-
SB-02	Female	Young	-	-	-	+	-	-
AL-04	Male	Adult	-	+	-	+	-	-
BA-02	Female	Young	-	-	-	+	-	-
BA-04	Female	Adult	-	-	-	+	-	-
TL-01	Female	Adult	-	+	-	+	-	-
TL-03	Female	Young	-	+	-	+	-	-

LB-01	Male	Adult	-	-	-	+	-	-
LB-02	Female	Adult	-	-	-	+	-	-
LB-03	Male	Adult	-	-	-	+	-	-
LB-04	Male	Adult	-	-	-	+	-	-
LB-05	Male	Adult	-	-	-	+	-	-
LB-06	Female	Young	-	-	-	-	-	+
LB-07	Female	Adult	-	-	-	+	-	-
LB-08	Male	Adult	-	-	-	+	-	-
CB-02	Male	Young	-	+	-	-	-	-
CB-03	Male	Young	-	+	-	-	-	-
CB-04	Female	Young	-	+	-	+	-	-
TR-01	Female	Young	-	-	-	+	-	-
TR-02	Male	Adult	-	+	-	+	-	-
TR-03	Female	Adult	-	+	-	+	-	-
TR-05	Female	Young	-	-	-	+	-	-
TR-06	Female	Adult	-	+	-	+	-	-
TR-09	Female	Adult	-	+	-	+	-	-

Table S3. Summary of co-infections between microeukaryote parasites and HEV and respective *p* value from wild boar of Portugal.

		HEV		***p*-value**
		**Positive**	**Negative**	
Cryptosporidium scrofarum	**Positive**	0 (0%)	2 (100%)	0.527
	**Negative**	4 (2.8%)	138 (97.2%)	
Balantioides coli	**Positive**	2 (9.5%)	19 (90.5%)	0.056
	**Negative**	2 (1.6%)	121 (98.4%)	
Giardia duodenalis	**Positive**	0 (0%)	0 (0%)	-
	**Negative**	4 (2.8%)	140 (97.2%)	
Blastocystis	**Positive**	0 (0%)	42 (100%)	0.198
	**Negative**	4 (3.9%)	98 (96.1%)	
Enterocytozoon bieneusi	**Positive**	0 (0%)	0 (0%)	-
	**Negative**	4 (2.8%)	140 (97.2%)	
